# Characterization and Fluctuations of an Ivermectin Binding Site at the Lipid Raft Interface of the N-Terminal Domain (NTD) of the Spike Protein of SARS-CoV-2 Variants

**DOI:** 10.3390/v16121836

**Published:** 2024-11-27

**Authors:** Marine Lefebvre, Henri Chahinian, Bernard La Scola, Jacques Fantini

**Affiliations:** 1 IHU Méditerranée Infection, 19-21 Boulevard Jean Moulin, 13005 Marseille, France; marinelfv@gmail.com (M.L.); bernard.la-scola@univ-amu.fr (B.L.S.); 2 Microbes Evolution Phylogeny and Infections (MEPHI), Aix-Marseille Université, 27 Boulevard Jean Moulin, 13005 Marseille, France; 3 Assistance Publique-Hôpitaux de Marseille (AP-HM), 264 Rue Saint-Pierre, 13005 Marseille, France; 4 Department of Biology, Faculty of Medicine, Aix-Marseille University, INSERM UA16, 13015 Marseille, France; henrichahinian@gmail.com

**Keywords:** ivermectin, antiviral, SARS-CoV-2, ganglioside, lipid raft, docking

## Abstract

Most studies on the docking of ivermectin on the spike protein of SARS-CoV-2 concern the receptor binding domain (RBD) and, more precisely, the RBD interface recognized by the ACE2 receptor. The N-terminal domain (NTD), which controls the initial attachment of the virus to lipid raft gangliosides, has not received the attention it deserves. In this study, we combined molecular modeling and physicochemical approaches to analyze the mode of interaction of ivermectin with the interface of the NTD-facing lipid rafts of the host cell membrane. We characterize a binding area that presents point mutations and deletions in successive SARS-CoV-2 variants from the initial strain to omicron KP.3 circulating in many countries in 2024. We show that ivermectin has exceptional flexibility, allowing the drug to bind to the spike protein of all variants tested. The energy of interaction is specific to each variant, allowing a classification according to their affinity for ivermectin in the following ascending order: Omicron KP.3 < Delta < Omicron BA.5 < Alpha < Wuhan (B.1) < Omicron BA.1. The binding site of ivermectin is subject to important variations of the NTD, including the Y144 deletion. It overlaps with the ganglioside binding domain of the NTD, as demonstrated by docking and physicochemical studies. These results suggest a new mechanism of antiviral action for ivermectin based on competitive inhibition for initial virus attachment to lipid rafts. The current KP.3 variant is still recognized by ivermectin, although with an affinity slightly lower than the Wuhan strain.

## 1. Introduction

Understanding the infection mechanisms of RNA viruses is essential for developing effective vaccine and therapeutic strategies. Typically, research in virology focuses on identifying host cell plasma membrane proteins that act as receptors for these viruses. Thus, CD4 has been identified as the main receptor for HIV-1 [1,2] and ACE2 that of SARS-CoV-2 [3]. This conception, widely shared by most virologists on the planet, however, ignores the fact that before interacting with a specific receptor, the virus must first be attracted by the cell and attach to its surface [4]. This step, which is the earliest in the infection cycle of RNA viruses, is generally bypassed in virus–host interaction studies. Thus, vaccine and therapeutic approaches remain focused on inhibiting the interaction between the virus and its receptor, either by antibodies (active or passive immunotherapy) or by molecules that are often repositioned when they prove to have an inhibitory effect on virus–receptor interactions. The initial interaction of an RNA virus with the host cell relies chiefly on an electrostatic attraction between the viral envelope proteins and lipid rafts present at the cell surface [5]. Due to their richness in gangliosides possessing negatively charged sialic acids, these rafts offer an electronegative landing platform for viruses whose envelope proteins have cationic zones [6]. The virus–membrane interaction can then be broken down into two steps according to the dual receptor model proposed thirty years ago by N. Yahi and J. Fantini for HIV-1 and, more recently, for SARS-CoV-2 [7]. In this model, the virus recognizes two distinct receptors, the raft gangliosides and a protein receptor. The cooperation of these two types of receptors then allows optimal adhesion of the virus to the surface of the host cell. The dual interaction is mediated by two distinct domains of the virus adhesion protein, thus allowing the formation of a ganglioside–virus–protein ternary complex [8,9,10,11]. The SARS-CoV-2 spike protein recognizes raft gangliosides by its N-terminal domain (NTD), whose flat surface has clearly been optimized during the evolution of this coronavirus and its probable passage through different animal hosts [11,12]. The ACE2 receptor is recognized by another region of the spike protein called the receptor binding domain (RBD) [13]. A key point of this mechanism is that the ganglioside binding domain of the NTD is immediately accessible, while the RBD remains cryptic until the virus lands on a raft. It is, therefore, crucial to develop therapeutic and vaccine approaches targeting the ganglioside binding domain of the NTD to block the initial attachment of the virus to the surface of the host cell. The immune response is generated by such antibodies that have a strong neutralizing power [14,15,16]. At the therapeutic level, we have previously shown that azithromycin binds to the ganglioside binding domain of the NTD of the spike protein (Wuhan B.1 strain) [17], which may legitimize its interest in anti-COVID-19 therapy, alone or in combination with other antivirals [18,19]. In this new study, which combines in silico and physicochemical approaches, we show that ivermectin, which is part of the therapeutic arsenal repositioned as an antiviral [20,21], recognizes the ganglioside binding domain of successive SARS-CoV-2 variants from the Wuhan B.1 strain to the KP.2/KP.3 variants [22,23,24,25] circulating in 2024. We characterize its binding site on the NTD of the different variants, and we demonstrate that its conformational flexibility allows it to adapt to all these variants.

## 2. Materials and Methods

### 2.1. Materials

Ivermectin was supplied by Sigma Aldrich (St. Quentin Fallavier, France). The stock solution was diluted in 2.5% DMSO and 97.5% water. Full-length trimeric recombinant spike proteins (BioServUK, Sheffield, UK) of four SARS-CoV-2 strains were used in this study: Wuhan, Alpha, Delta, and Omicron BA.1 (BSV-COV-PR-33, BSV-COV-PR-65, BSV-COV-PR-97, and BSV-COV-OM-0.1, respectively). The spike protein of each viral strain was dissolved in PBS at a concentration of 2 nM and tested at a final concentration of 5 pM in the monolayer assay.

### 2.2. Molecular Modeling Simulations

In silico analyses were performed using the Hyperchem and Molegro Molecular Viewer as described previously [10]. Interaction energies were calculated from stable complexes using the Ligand Energy Inspector function of Molegro. A complete structure of the reference spike protein was generated from pdb 7bnm, as previously described [26]. All gaps in the pdb file were fixed by inserting the missing amino acids with the protein structure prediction service Robetta, https://robetta.bakerlab.org/ (accessed on 10 August 2024) [27]. This source file model was used to introduce the specific mutational profiles of the indicated Alpha, Delta, and Omicron variants with the MUTATE tool of the Swiss-Pdb Viewer [28]. The trimeric structure in the closed pre-fusion conformation was constructed using the Swiss-Pdb Viewer by homology with a reference model (pdb: 6VSB). All structures were then submitted to several rounds of energy minimization with the Polak–Ribière algorithm. The electrostatic surface potential was analyzed using the Molegro Molecular Viewer [29]. The docking of ivermectin on the spike proteins was performed using Hyperchem [30,31,32,33] as previously described [17]. Several initial conditions were tested, and only those with the highest energy interaction after energy minimization with the Polak–Ribière algorithm were selected. The energy of interaction of each molecular complex was calculated using the Ligand Energy Inspector tool of the Molegro Molecular Viewer [34]. A detailed description of our flexible docking method, compared with other approaches, is provided at the beginning of Section 3.

### 2.3. Langmuir Microtensiometry

Surface pressure measurements were performed using the Langmuir film balance technique with a fully automated microtensiometer (µTROUGH SX, Kibron Inc., Helsinki, Finland). The interaction of a peptide (or a protein) with a ganglioside monolayer is an interfacial phenomenon that can be studied by surface pressure (π) measurements [35,36,37]. The interaction of a protein with a ganglioside lipid monolayer can be detected at a constant area by an increase in the surface pressure (Δπ). This increase is caused by the insertion of the protein between the polar heads of vicinal gangliosides, which is not counterbalanced by an increase in the area of the monolayer. This effect can be followed kinetically by real-time surface pressure measurements after injecting the protein into the aqueous subphase underneath the ganglioside monolayer, as described previously [38]. The initial velocity of the insertion process is expressed as mN·m^−1^·min^−1^. All experiments were conducted at 20 °C in triplicate. To test the inhibitory effect of ivermectin, the drug was preincubated with the spike proteins for 30 min at room temperature before injection underneath the ganglioside monolayer.

## 3. Results

### 3.1. Flexible Docking Analysis

Due to the rapid development of artificial intelligence (AI) these last years, molecular docking can now be used by researchers who may not have any prior experience in this field, whatever their background in structural biochemistry. In this case, step-by-step procedures are available, allowing anybody to generate and download ligand–protein complexes at the atomic scale [39]. However, the automatic docking methods offered by software such as Dock [40] or Autodock [41] suffer from significant limitations which can generate artifacts that can partially or even completely call into question the results obtained [42,43,44,45]. Thus, one of the critical elements of molecular docking is taking into account the flexibility of proteins at the level of amino acid side chains but also at the level of their three-dimensional structure, in particular when loops with no stable structure are involved in the ligand binding site [46]. Often, this site does not pre-exist on the protein and is gradually created during the interaction with the ligand, according to an induced fit process widely described in the literature [47,48,49,50], as is the case for intrinsically disordered proteins (IDPs) [51,52,53]. It is, therefore, crucial to validate the docking results a posteriori by molecular dynamics (MD) or molecular mechanics (MM) simulations, which follow the evolution of a protein–ligand complex obtained by automatic docking [43,54,55,56,57,58]. Thus, many studies are carried out according to a two-step protocol, starting with a molecular docking study and followed by MD or MM simulations. This approach allows us to validate or invalidate the docking results by following the protein–ligand complex evolution over time [43]. The simulation time then becomes a new critical parameter of the method. In 30 years, it has gone from a few picoseconds to a hundred nanoseconds, and simplifications such as coarse grain now make it possible to reach the millisecond scale for complex systems, including, for example, a cell membrane [59,60,61,62].

The strategy that we have developed for many years in our team is different. Rather than using rigid docking conditions (protein and ligand immobilized in starting structures that do not evolve during the simulation) or semi-flexible (taking into account the conformational freedom of the amino acid side chains on a fixed secondary structure), our approach takes into account the conformational flexibility of the protein and the ligand without a priori. The simulation is carried out using the Polak–Ribière conjugate gradient method [63,64,65,66], which allows the energy minimizations of the two partners to be obtained before the interaction and throughout the binding process [38,67]. The flexibility of the ligand and its adaptation to its binding site obey dynamic molecular mechanisms that are fully considered by our method. This approach allows for the combination of docking, flexibility of the protein and the ligand, and evolution of the complex as a function of time until stable conditions are reached. The computation time, although faster than MM and especially MD simulations, remains high for systems with several thousand atoms, such as those analyzed in the present study [68]. The Polak–Ribière energy minimization is an iteration of cycles until the system reaches a root-mean-square (RMS) gradient of 0.1 kcal· Å^−1^·mol^−1^ as the convergence condition. The robustness of the complex can then be confirmed by further lowering this threshold, which we performed in our docking study of ivermectin on the spike protein of SARS-CoV-2 variants (convergence condition of 0.01 kcal· Å^−1^·mol^−1^).

### 3.2. Mutational Landscape of the Spike Protein from the Original B.1 Strain to KP.3

**Figure 1:**
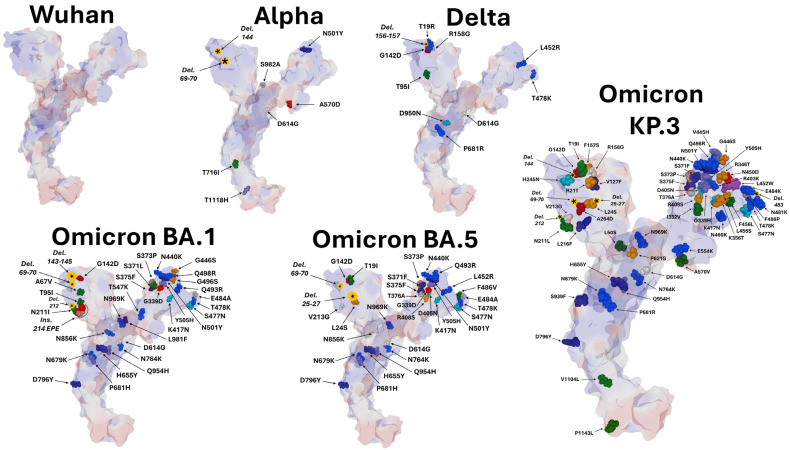
Mutational landscape of SARS-CoV-2 spike proteins. The electrostatic surface potential of the protein is visible by transparency. Amino acid residues are represented in atomic spheres and colored according to the amino acid type (e.g., acidic residues are colored in red). Deletions and insertions are indicated by an asterisk in a yellow disk. Legend: Wuhan, B.1; Alpha, B.1.7; Delta, B.1.617.2.

The evolution of the spike protein from the original B.1 strain to the KP.3 variant circulating in 2024 is characterized by a progressive accumulation of mutations and indels (insertions/deletions) (Figure 1). Despite this accumulation of mutations in the NTD and RBD functional domains, culminating in the omicron series variants, the virus maintained its ability to bind to raft gangliosides and to the ACE2 receptor. However, this large structural variability requires specific studies for each variant. We, therefore, began by searching for a potential ivermectin site on the NTD of strain B.1, and then we studied the evolution of this site on the alpha, delta, and omicron variants shown in Figure 1.

### 3.3. Characterization of an Ivermectin Binding Site in the NTD of the B.1 Spike Protein

**Figure 2:**
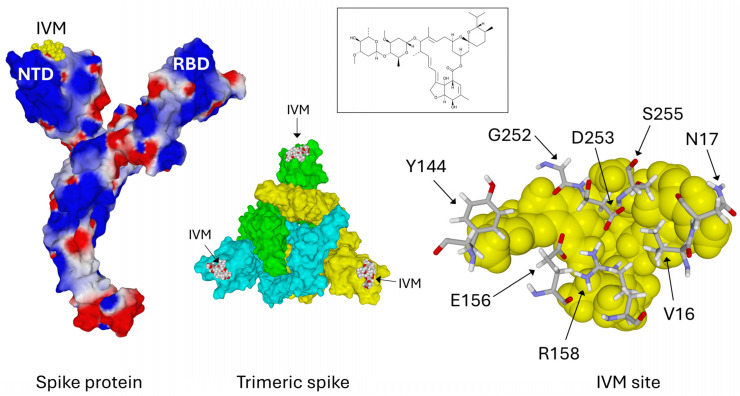
Characterization of an ivermectin binding site on the NTD of the B.1 spike protein. Left panel: electrostatic surface potential of the spike protein (electronegative areas in red, electropositive areas in blue, neutral areas in white). NTD, N-terminal domain; RBD, receptor binding domain; IVM, ivermectin (atomic spheres colored in yellow). Central panel: trimeric spike protein, each subunit in a different color (cyan, green, and yellow), and ivermectin bound to each NTD (in atoms colors, with oxygen in red). In this prefusion conformation, the central RBDs are still masked and not available for ACE2. Right panel: key amino acid residues of the B.1 NTD determining the ivermectin binding site. For clarity, Q14 is not represented. Ivermectin is represented in atomic spheres and colored yellow. Its chemical structure is shown in the central inset.

Our molecular modeling studies have previously identified a binding site for raft gangliosides at the top of the NTD in a large flat area. Here, we present a new docking study for ivermectin, based on the same molecular modeling principles with minimized spike protein structures extracted from the Protein DataBank files and completed for all gaps. This allowed us to identify an ivermectin binding site also located on the NTD surface (Figure 2).

The total energy of interaction between ivermectin and the B.1 NTD complex is −129.5 kJ·mol^−1^ (Table 1). The trimeric spike protein has three NTDs, and it can thus accommodate three ivermectin molecules simultaneously.

**Figure 3:**
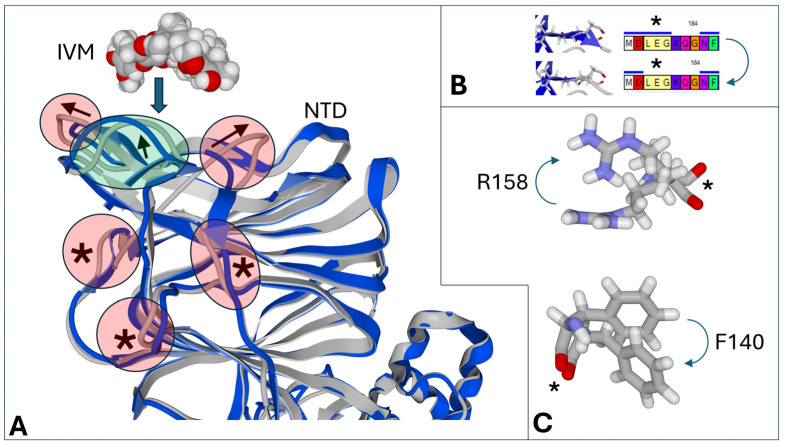
Flexible docking of ivermectin (IVM) on the spike NTD. (**A**) Movements of the secondary structure of the spike protein during the docking of ivermectin (IVM) on the NTD of the Wuhan spike protein. The initial secondary structure is colored blue, and the final structure is grey. The movements involving the ivermectin binding site are indicated by arrows. Long-range conformational changes are indicated by asterisks. (**B**) Loss of a part of a beta-strand during the simulations (arrow). The upper sequence corresponds to the initial structure with amino acid residues 179-LEG-181 belonging to a beta-strand, and the lower sequence to the final structure with the same amino acids excluded from the beta-strand. (**C**) Flexibility of amino acid chains upon docking (arrows). Movements of the secondary structure are indicated by an asterisk. R158 belongs to the ivermectin binding site but not F140.

The models in Figure 3 illustrate the robustness of our docking method. The superposition of the spike protein structure before and after docking shows significant differences in the secondary structure (Figure 3A,B) and in the side chains interacting with the ligand (R158) or much more distant (F140) (Figure 3C), which indicates a good consideration of long-range conformational changes that are generally ignored by classical automatic docking techniques.

Figure S1 shows the evolution of the complex formed by a spike protein and ivermectin. This animation shows the movements of the entire protein (including the RBD) during the interaction with the ligand, which perfectly illustrates our all-atom flexible docking method.

### 3.4. The Ivermectin Binding Site Occupies a Large Part of the GM1-Binding Domain of the NTD

**Figure 4:**
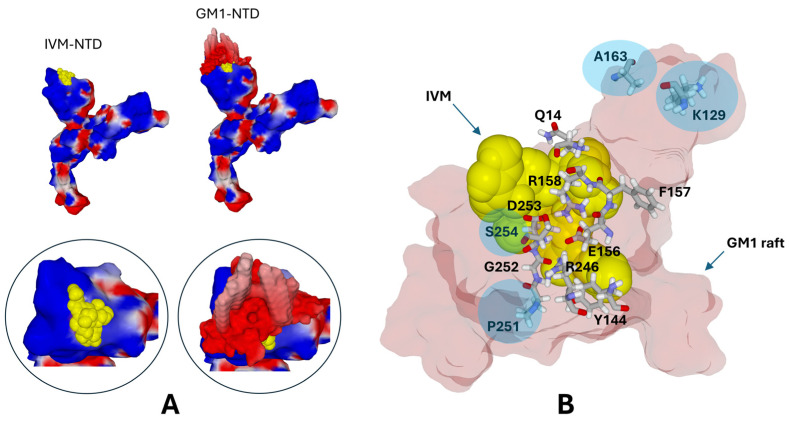
Ivermectin occupies a large part of the GM1-binding domain of the NTD. (**A**) Structural models of the NTD in complex with ivermectin (IVM) or a GM1 raft. Ivermectin is represented in atomic yellow spheres. The spike protein (Wuhan strain) and the GM1 raft are represented in electrostatic surface potential (hence mostly red for the GM1 raft, i.e., electronegative, and red/blue/white for the spike protein, as in Figure 2). The insets show the molecular complexes at higher magnification. Ivermectin is superposed to the GM1 raft to visualize the respective locations of both binding sites. (**B**) Superposition of the ivermectin (IVM, atomic yellow spheres) and GM1 raft (light pink surface rendition) binding sites on the spike NTD. The main amino acid residues involved in both binding sites are Q14, E156, R158, G252, D253, and R246. F157 is close, but it contributes only marginally to the binding of ivermectin. Residues indicated with a blue disk are critical for GM1 binding but outside the ivermectin binding site.

We have previously published the molecular characteristics of the interaction between a GM1 ganglioside raft and the isolated NTD of the spike protein [17]. In the present article, we studied the interaction of a similar GM1 raft with the NTD of the entire spike protein. This new simulation allowed us to clarify how the spike protein interacts with the GM1 ganglioside cluster and to compare the results with the spike–ivermectin docking (Figure 4). It appears that ivermectin occupies a significant part of the raft binding site. Many amino acids participate in both complexes, including Q14, E156, R158, G252, D253, and R246. These results strongly suggest that ivermectin can act as a competitive inhibitor of the raft–NTD interaction.

### 3.5. Structural Variations of the Ivermectin Binding Site in the NTD of SARS-CoV-2 Variants

**Figure 5:**
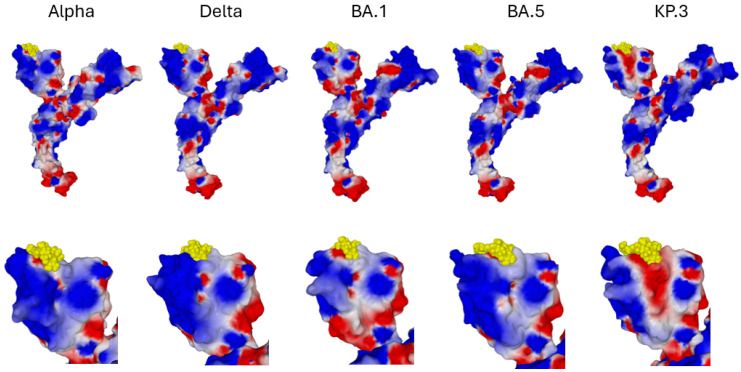
Structural evolution of the ivermectin binding site on the NTD of the alpha, delta, and omicron variants. Upper panels: For each variant, the whole spike protein is shown in complex with ivermectin (represented in atomic spheres and colored in yellow). The spike protein is represented as in Figure 2 (electrostatic surface potential). Lower panels: magnification of the NTD–ivermectin complex illustrating the different modes of interaction of the drug according to the variant studied. Note that the NTD varies both in global shape and in electrostatic surface potential.

In the second step, we analyzed the interaction of ivermectin with the different variants of SARS-CoV-2 (Figure 5). Despite significant structural variations due to the accumulation of mutations and indels in the NTD, it appears that ivermectin is able to bind to all variants. However, the modalities of this interaction vary greatly depending on the variants, which affects the affinity of ivermectin (Table 1).

A complete analysis of the interaction modalities of ivermectin with the different spike proteins is presented in Table 1. It emerges from this analysis that ivermectin has surprising adaptive capacities in regard to the evolution of spike proteins. Thus, the amino acids involved in the interaction vary for each complex, both in terms of their absence or presence on the site but also in terms of the interaction energy of each amino acid. For instance, R19 appears only in the complex with the NTD of the Delta variant. Y144 is involved in the binding of ivermectin to Wuhan and Delta spikes. S254 is important for the binding of ivermectin to the NTD of Omicron BA.1 and BA.5 variants. Four amino acids are significantly involved in the interaction of ivermectin with all variants: Q14, V16, N17, and D253, with various values of the energy of interaction.

**Table 1:** Evolution of ivermectin binding site in the NTD of SARS-CoV-2 variant spike proteins.

Amino Acid	Wuhan B.1	Alpha B.1.1.7	Delta B.1.617.2	Omicron BA.1	Omicron BA.5	Omicron KP.3
**Q14**	−10.0	−10.6	−13.7	−19.4	−19.4	−13.4
**C15**	−7.8	−7.7	−2.5	−6.4	−6.4	−9.2
**V16**	−19.3	−15.4	−17.1	−16.4	−16.4	−18.9
**N17**	−8.3	−10.6	−8.1	−8.4	−8.4	−7.6
**R19**	(-)	(-)	−4.4	(-)	(-)	(-)
**Y144**	−11.9	(-)	−5.9	(-)	(-)	(-)
**H146**	(-)	−7.0	−2.4	−2.5	−2.5	(-)
**K147**	(-)	−20.8	(-)	−8.6	−8.6	−1.3
**E156**	−5.6	−9.5	(-)	−12.9	−12.9	−11.2
**R158**	−13.1	−0.3	(-)	−10.3	−10.3	(-)
**R246**	−7.3	(-)	−3.0	−16.2	−16.2	−6.4
**G252**	−6.0	−3.3	−2.7	−1.9	−1.9	−1.9
**D253**	−29.1	−23.6	−28.5	−25.9	−25.9	−17.1
**S254**	−0.8	−0.3	−2.1	−18.2	−18.2	−0.7
**S255**	−4.1	−5.8	−8.8	−3.0	−3.0	−5.1
**Total**	−129.5	−125.1	−107.0	−159.4	−121.6	−101.5

Energy values are calculated from molecular models of ivermectin bound to the indicated spike protein in Figure 2 and Figure 5 and expressed as ΔG in kJ·mol^−1^. For the sake of clarity, only amino acids contributing more than 5 kJ·mol^−1^ in absolute value are mentioned, except when the concerned amino acid has a ΔG > 5 kJ·mol^−1^ for another variant. (-) indicates that the amino acid is not involved in ivermectin binding (either because it is deleted in the corresponding variant or because it is expressed but does not belong to the ivermectin binding site).

**Figure 6:**
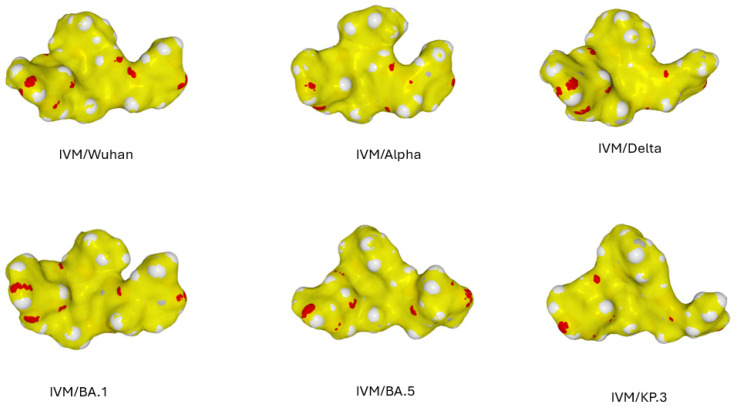
Different conformations adopted by ivermectin in complex with each NTD variant. The molecule is represented in atomic spheres superimposed with a slightly transparent surface colored in yellow. Note that the molecule can adopt various “octopus-like” conformations (a central head and lateral arms) according to the spike protein with which it interacts. An animation (Figure S2) with a superposition of all these conformers illustrates the high flexibility of ivermectin.

This structural analysis, therefore, makes it possible to classify the different variants according to their affinity for ivermectin in the following ascending order: Omicron KP.3 < Delta < Omicron BA.5 < Alpha < Wuhan (B.1) < Omicron BA.1. What is striking about this classification is that it does not absolutely follow the chronological order of appearance of the variants. The NTD of the original Wuhan strain (B.1) is in the high part of the ranking, with an interaction energy of −129.5 kJ·mol^−1^. The highest affinity is reached by Omicron BA.1 (−159.4 kJ·mol^−1^), the lowest by Omicron KP.3 (−101.5 kJ·mol^−1^). Regardless, there is a remarkable structural adaptation of ivermectin to the NTD of each variant. This can only be explained by the conformational flexibility of ivermectin, illustrated in Figure 6. These different conformations recall the movements of an octopus with a central head and lateral arms. This is particularly well illustrated in the animation of Figure S2.

### 3.6. Ivermectin Inhibits the Binding of SARS-CoV-2 Spike Trimers to Gangliosides

**Figure 7:**
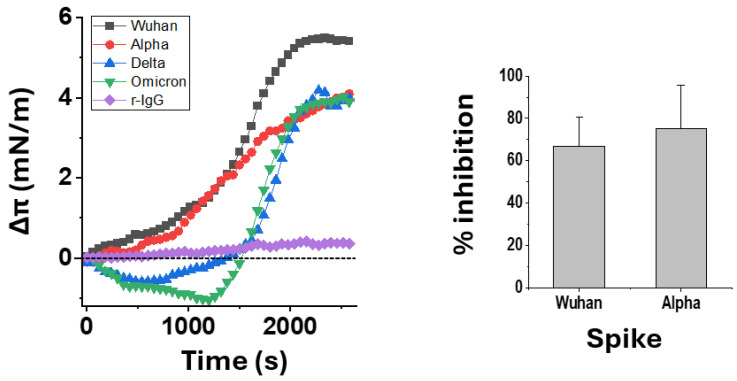
The interaction of trimeric spike proteins with a monolayer ganglioside GM1 is inhibited by ivermectin. Left panel: kinetics of interaction of the indicated spike protein trimer (Wuhan, B.1; Alpha; Delta; and Omicron BA.1, tested at a final concentration of 5 pM) with a GM1 monolayer prepared at an initial pressure of 20 mN·m^−1^. For clarity, a representative experiment performed on the same days with the 4 spike proteins is shown (the experiment was repeated three times with similar results). Rabbit IgG (r-IgG) was used as a negative control. Right panel: preincubation of the Wuhan B.1 and Alpha spike proteins (5 pM) with ivermectin (800 nM) resulted in a reproducible inhibition of the initial velocity of the interaction calculated during the first 10 min of the incubation with the monolayer (calibrated against a control experiment with the ivermectin vehicle only considered as 100%). Results are expressed as mean % of inhibition ± SD (*n* = 3).

To validate these in silico studies, we performed a physicochemical analysis of the interaction of the spike protein with the GM1 ganglioside monolayer. In these experiments, the trimeric recombinant spike protein of each variant is injected into the aqueous phase underneath the ganglioside monolayer. Its interaction with the gangliosides is monitored in real-time by surface pressure measurements (Figure 7), showing the interaction kinetics of the spike proteins B.1, Alpha, Delta, and Omicron BA.1 with the GM1 ganglioside monolayers.

The four spike proteins interacted well with GM1 but with different modalities that reflect their structural differences. The spikes of the Wuhan B.1 strain and the Alpha variant seem to interact directly with the GM1 monolayer without any latency. In contrast, the spikes of Delta and Omicron BA.1 first affect the surface pressure downward before it increases, as in the case of the other two spike proteins. Overall, these increases in surface pressure, with or without latency, unambiguously indicate an interaction with GM1, thus confirming the structural studies carried out in silico. Indeed, an irrelevant protein (rabbit IgG) tested in similar conditions underneath a GM1 monolayer did not induce any surface pressure change. In the second step, we preincubated the Wuhan B.1 and Alpha spike proteins with ivermectin and then measured the impact of this treatment on the initial velocity of the spike–GM1 interaction. It is important to note that this experiment could not be performed with the Delta and Omicron BA.1 spikes due to the latency time observed with these two spike proteins. On the other hand, the initial speed of interaction is a critical parameter of viral infection; any drug capable of delaying this interaction could potentially block viral infection at its earliest stage. The results in Figure 7 show that ivermectin is able to inhibit the spike–GM1 interaction, in agreement with molecular docking studies that have identified an ivermectin binding site in the middle of the ganglioside binding domain of the NTD.

## 4. Discussion

Our team uses a molecular docking method that takes into account the flexibility of the protein and the ligand before and during the formation of the protein–ligand complex. This approach eliminates the artifacts often encountered in automatic docking studies, which then require the implementation of computationally time-consuming simulations such as MDS or MMS. On many occasions, we have validated the predictions obtained by our method using experiments, in particular by introducing mutations into the protein that lower the interaction energy of the protein–ligand complex [69]. We have also subjected several of our docking results to MDS, thus obtaining a double validation of our method [17]. Regarding ivermectin, it is worth noting an artifact encountered in automatic docking for molecules with rings. Automatic docking often considers these rings as aromatic structures, while ivermectin does not have any. Thus, the docking of ivermectin on the RBD domain of the spike protein can be carried out with an unrealistic planar structure of the ligand, which requires additional MDS simulations to relax the ivermectin molecule and allow it to adopt more realistic conformations [70]. Our own search for an ivermectin binding site on the RBD of the spike protein confirmed the results obtained in this direction by another team [71]. However, the interaction energy of the RBD–ivermectin complex is lower than that of the NTD-IVM complex (Figure S3). We therefore focused on the NTD, as this region has been previously shown to contain a sialic acid binding domain, which may confer ganglioside recognition [8,72]. The NTD also has the particularity of being immediately accessible on the surface of the spike protein trimers carried by the virus, whereas the RBD must be unmasked to interact with its ACE2 receptor in a typical dual receptor mechanism dependent on the initial NTD–ganglioside interaction [9,73]. Finally, there are three IVM binding sites (one at each end of the NTD) on the trimer, which is compatible with the inhibitory effect of IVM on hemagglutination [74]. Overall, it is clear that the NTD is a promising target for designing antibodies and small molecules able to hamper the SARS-CoV-2 attachment to host cells [73].

In the present study, we identified a binding site for ivermectin at the surface of the NTD of the SARS-CoV-2 spike protein. This site was first characterized on the spike protein of the original Wuhan B.1 strain. The involvement of the amino acid Y144 could suggest that this site no longer existed in SARS-CoV-2 variants with the Y144 deletion, especially Alpha [26] and Omicron BA.1 [75]. Furthermore, it appears that these two spike proteins have found two distinct paths to reconstitute the ivermectin binding site since the measured interaction energies are different (in particular, note the respective contributions of Q14, R246 and S254 in Table 1). It can be considered that the selection pressure that has allowed the preservation of a functional interaction with the raft gangliosides has also allowed the preservation of a good affinity for ivermectin. This is manifested by interaction energy with the current variant KP.3 (−101.5 kJ·mol^−1^) close (78%) to that of the original strain (−129.5 kJ·mol^−1^), despite the accumulation of 15 mutations and deletions in the NTD and more than 60 in the whole spike protein [22]. In a previous study, we compared raft gangliosides to quicksand, in which the spike protein NTD engages and is then stabilized by a network of hydrogen bonds [6]. This mechanism is possible because raft gangliosides have great conformational freedom, especially at the periphery of the rafts, which is a privileged place for interaction with many ligands [76]. As for ivermectin, we find great flexibility inherent in the molecule itself, which allows it to adapt to the different spike proteins of the variants and mimic the quicksand effect of rafts. In this case, we can describe the ivermectin molecule as a very flexible structure that can deform in all directions of space like an octopus (Figure 6 and Figure S2). Incidentally, this mechanism is fundamentally opposed to the classical concept of the key and lock in molecular interactions [77]. This exceptional flexibility may allow additional interactions of ivermectin with the RBD of SARS-CoV-2 [71]. In this respect, conformational flexibility, which finely controls virus–receptor interactions [78,79], is a critical issue that needs to be considered in docking studies of potential antiviral drugs [80].

Our modeling results were complemented by physicochemical experiments demonstrating the interaction of trimeric recombinant spike proteins with ganglioside monolayers mimicking the raft surface exposed to virus NTDs. We could test the Wuhan, Alpha, Delta, and Omicron BA.1 spikes that are commercially available for experimental research. In the future, it will be interesting to test other recombinant spike proteins, such as KP.3. We were also able to test the effect of ivermectin on the interaction of the Wuhan and Alpha spike proteins with GM1 monolayers. In these two cases, which were chosen because they did not present any delay in the interaction kinetics (Figure 7), we demonstrated an inhibitory effect of ivermectin. These physicochemical studies validate the results obtained in silico because the site identified for ivermectin is right in the middle of the ganglioside binding domain of the NTD. In these experiments, the excess of ivermectin relative to the protein was 1600-fold. This may seem high, but on the one hand, this ratio is in line with the concentrations of ivermectin necessary to inhibit the hemagglutination induced by spike proteins [74] and on the other hand, ivermectin is an amphipathic compound that is only really soluble below 300 nM. Above this threshold value, which corresponds to its critical micellar concentration, ivermectin forms micelles and other molecular aggregates whose biological activity remains undetermined. Finally, there are three ivermectin binding sites per trimeric spike, which further decreases the molecular drug/spike ratio to 533, and 200 if we consider only the monomeric ivermectin molecules in solution above a concentration of 300 nM. Thus, our results support the idea that ivermectin behaves as a competitive inhibitor of the attachment of spike protein trimers to raft gangliosides. This mechanism of action, identified for the first time, could explain the antiviral effects of ivermectin on SARS-CoV-2 infection [81], and on the hemagglutination induced by the spike proteins of different SARS-CoV-2 variants [74,82,83,84]. In both cases, blocking the spike–ganglioside interaction could explain the beneficial effects of ivermectin, which deserves further evaluation for the potential treatment of COVID-19 disease and related symptoms.

## Data Availability

The raw data supporting the conclusions of this article will be made available by the authors on request.

## Supplementary Materials

The following supporting information can be downloaded at https://www.mdpi.com/article/10.3390/v16121836/s1; Figure S1: Evolution of the complex formed by a spike protein and ivermectin in flexible docking; Figure S2: Conformational flexibility of ivermectin; Figure S3: Docking of ivermectin on the receptor-binding domain (RBD) of the Wuhan spike protein.
