# Supplementary material for: Characterization and Fluctuations of an Ivermectin Binding Site at the Lipid Raft Interface of the N-Terminal Domain (NTD) of the Spike Protein of SARS-CoV-2 Variants
Source: Viruses. 2024 Nov 27;16(12):1836. doi: 10.3390/v16121836 (PMC11680242; doi:10.3390/v16121836)

***Supplementary Material***

Characterization and fluctuations of an ivermectin binding site at the lipid raft interface of the N-terminal domain (NTD) of the spike protein of SARS-CoV-2 variants.

Marine Lefèbvre^1,2^, Henri Chahinian^2^, Bernard La Scola^1^ and Jacques Fantini^2^

^1^IHU Méditerranée Infection, 19-21 Boulevard Jean Moulin, 13005 Marseille, France; Aix-Marseille Université, Microbes Evolution Phylogeny and Infections (MEPHI), 27 Boulevard Jean Moulin, 13005 Marseille, France; Assistance Publique-Hôpitaux de Marseille (AP-HM), 264 Rue Saint-Pierre, 13005 Marseille, France.

^2^Aix-Marseille University, Department of Biology, Faculty of Medicine, INSERM UA16, Marseille, France.

Correspondance: jacques.fantini@univ-amu.fr

**Figure S1. Evolution of the complex formed by a spike protein and ivermectin in flexible docking.** This animation illustrates the all-atom flexible docking method (both protein and ligand) used in this study. The spike protein (Alpha variant) is represented in surface rendition of the electrostatic potential (red, electronegative; blue, electropositive; white, neutral). Ivermectin is in yellow atomic spheres.


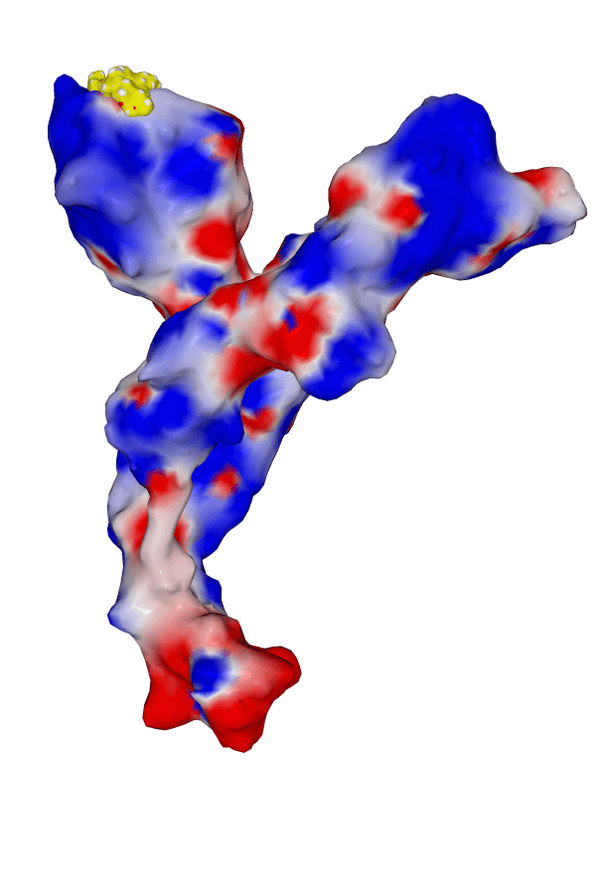


**Figure S2. Conformational flexibility of ivermectin.** This animation shows a superposition of the different conformations of ivermectin bound to each spike protein variant (from Wuhan to Omicron KP.3). Ivermectin is represented in atomic spheres superimposed with a slightly transparent surface colored in yellow.


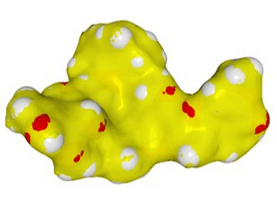


**Figure S3. Docking of ivermectin on the receptor-binding domain (RBD) of the Wuhan spike protein.** Two symmetric views of the complex are shown. The spike protein is represented in surface rendition of the electrostatic potential (red, electronegative; blue, electropositive; white, neutral). Ivermectin is in yellow atomic spheres. The values of the energy of interaction of individual amino acid residues involved in the complex are listed on the left panel.


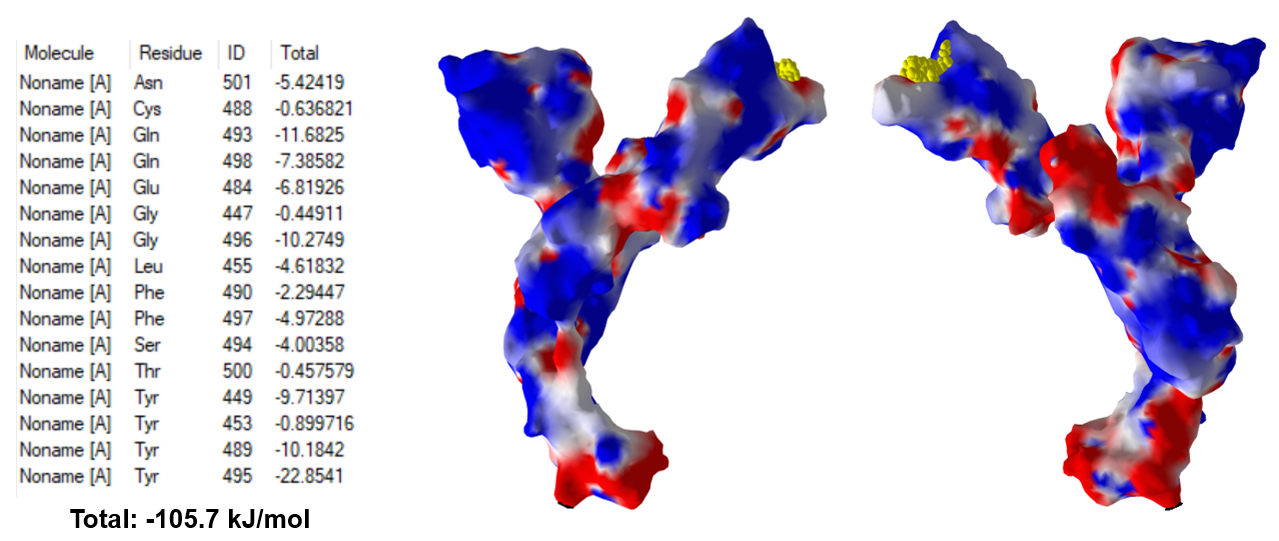

Supplement: Supplementary file 1 [file viruses-16-01836-s001.zip › viruses-3286012-supplementary.docx]
